# Supplementary material for: Are carbon emissions trading and green financial instruments synergistic? -Comprehensive quantitative research based on content analysis
Source: PLoS One. 2024 Mar 7;19(3):e0298601. doi: 10.1371/journal.pone.0298601 (PMC10919601; doi:10.1371/journal.pone.0298601)
Supplement: S1 Data — (ZIP) [file pone.0298601.s001.zip › Data/地级市Z_F工作报告.zip!/║╙─╧/╔╠╟≡╩╨/└┤╫╘╣1⁄2╓┌║┼í╛┬φ┐╦╩2╛▌═°í┐_2016.docx]

各位代表：

现在，我代表市人民政府，向大会报告政府工作，请予审议，并请各位政协委员和其他列席人员提出意见。

一、2015年及“十二五”工作回顾

2015年，面对经济下行压力，在省委、省政府的正确领导下，我市以党的十八大和十八届三中、四中、五中全会精神为指导，认真贯彻落实习近平总书记系列重要讲话精神，按照“持续求进，好中求快”总基调，统筹做好稳增长、促改革、调结构、惠民生各项工作，全市经济总体呈现缓中趋稳、稳中向好态势，较好完成了年初确定的目标任务。全市生产总值完成1803.9亿元，比上年增长8.7％；规模以上工业增加值完成491.4亿元，增长9.3％；固定资产投资完成1410.1亿元，增长14.3％；社会消费品零售总额完成666.2亿元，增长13.1％；一般公共预算收入完成110.7亿元，增长9.9％；城乡居民人均可支配收入分别增长8.4％、9.7％。

（一）现代产业体系逐步完善。一是加强主导产业培育。建立工业经济运行监测及中小企业服务综合平台，设立工业企业应急还贷周转金，先后举办纺织服装制鞋、电动车、食品、制冷装备和阿里巴巴·商丘产业带线上线下联动展会，着力破解资金、市场、土地、用工等难题。装备制造、食品工业、纺织服装制鞋等主导产业进一步壮大，电子信息、环保装备等新兴产业得到快速发展。规模以上工业企业实现主营业务收入2140亿元，增长12％。食品工业、服装服饰、现代家居、汽车及零部件、电子信息、装备制造六大高成长产业完成工业增加值282亿元，占规模以上工业比重达57.4％。高新技术产业增加值比上年提高4.7个百分点，高耗能产业增加值下降1个百分点。二是加快现代服务业发展。突出发展商贸物流业，加快豫东综合物流集聚区建设，商丘保税物流中心获批，电商产业园、大学生创业园、传化物流港开工建设。围绕火车站区域升级改造和高铁商城建设，打造市区北部商业圈，全市市场商品交易总额689.7亿元，增长13.5％。积极发展文化旅游业，全年接待游客1077万人次，增长12％；实现旅游收入22亿元，增长13％。大力发展金融服务业，交通银行、中信银行成功进驻商丘，新增村镇银行1家、保险机构4家、证券机构3家。科迪乳业在深交所上市，亚太能源、爱己爱牧、东和设备在新三板挂牌。全市金融机构各项存款余额1978.1亿元，增长12.5％；各项贷款余额1137.8亿元，增长13％。大力发展电子商务，阿里巴巴·商丘产业带已入驻企业1018家，累计交易额突破10亿元。全市电商企业1300余家。快递业完成2307万件，增长84.6％，业务收入增长56.9％。电子信息产业实现销售收入46亿元，成功建成全国首个百万级“全光网城市”。三是加快产业发展平台建设。加大基础设施建设和项目引进力度，强力推进科技创新、公共服务，全市产业集聚区和服务业“两区”建成区面积达107.6平方公里，从业人员35万以上。产业集聚区规模以上工业企业达到699家，增长37.3％；完成工业增加值407.2亿元，增长15％。澳柯玛冷藏车、欧莱克斯制冷设备、志永达织造、利斯特纺纱及服装面料、拉多美化肥、晶冠宇电子、金马药业等一批主导产业项目先后落地，民权县国家冷冻冷藏设备质检中心建成投用，成功创建国家级出口制冷机电产品质量安全示范区。

（二）农业生产持续稳定。一是稳定提高粮食生产能力。大力实施高标准粮田“百千万”工程，新建高标准粮田75万亩，全市粮食总产111.6亿斤（不含永城），实现“十二连增”。二是调整农业结构。大力发展果蔬花卉种植等高效农业，建设现代农业生产基地、农业标准化示范园区和都市农业工程，宁陵县成功申报国家农业科技示范区，夏邑县、民权县成功创建国家级出口食品农产品质量安全示范区。推动奶牛、生猪、家禽、肉牛肉羊集群发展，加快建设标准化、规模化生产基地，全年肉类总产量44.7万吨，禽蛋产量24.8万吨，奶类产量28.2万吨。三是培育现代农业产业化集群。积极推进现代农业产业园区、农产品集中区和农产品市场体系建设，市级以上龙头企业达252家，销售收入超亿元的88家，销售收入10亿元以上的产业化集群8个。健全土地流转机制，农业产业化经营主体8125家。四是加强农业基础建设。投入水利建设资金13.2亿元，实施任庄及马楼水库除险加固、中小河流治理、小农水重点县、灌区续建配套与节水改造工程，新增和改善灌溉除涝面积149万亩。

（三）城乡建设步伐加快。全市城镇人口354.2万人，城镇化率47％。一是强力推进中心城区建设。《商丘市城市总体规划（2015—2030）纲要》已经完成。以高铁商贸区、商务中心区、古城文化区三大区域建设为重点，统筹推进道路、管网、绿化等基础设施建设。新建、打通和升级改造城区道路100条，完成6条河道清淤贯通。开工建设汉梁文化公园、华夏游乐园，完成了火车站南广场、火车南站广场、金世纪广场升级改造，中心城区新增绿化面积338万平方米。继续开展“六城”联创，深入推进省级文明城、全国卫生城创建工作，巩固国家旅游城、园林城创建成果，国家森林城市创建基本达到验收标准，完成全国双拥模范城验收工作。先后开展了户外广告和门头牌匾治理、通信电力线路规范整治、三轮车电动四轮车专项治理。促进房地产市场平稳健康发展，实施古城拆迁补偿货币化安置政策，出台购买商品住房财政补贴政策，打通商品房、安置房和保障房转换渠道。二是大力发展县城组团。按照“改造老城、发展新区”的工作思路，加快县城新城区、产业集聚区、商务中心区和特色商业区等功能区开发，推进旧城改造步伐，增强县城辐射带动能力。三是有序推进小城镇建设。24个区位优势好、产业基础强的小城镇建设不断加快，交通、通信、文化、卫生、物流快递等功能向重点镇延伸。4个镇入选全省经济发达镇行政管理体制改革试点。四是改善农村人居环境。持续推进“美丽商丘·整洁村镇”活动，完成了3160个行政村整治任务，占全市行政村的82％，我市被定为全省改善农村人居环境示范市。

（四）改革创新不断深化。全市44大项201小项改革任务，已完成95小项，正在实施74小项。一是扎实推进政府机构改革。制定政府权力清单、责任清单和负面清单，清理行政审批事项97项，保留111项，市政府42个部门的权力清单全部编制公布，县（区）权力清单编制工作基本完成；有序推进工商、质监、食品药品监管等部门职责机构整合，积极推进“三证合一”商事制度改革；深化供销体制改革；交通运输行政执法体制改革全面完成。二是完善财税管理体制。认真落实新预算法，加强政府性债务管理，推进政府和部门预决算公开，建立跨年度预算平衡机制，完成了公务用车制度改革，确保了全市“三公”经费支出只减不增。三是统筹推进各项改革。扎实推进不动产登记制度改革和公共资源交易管理体制改革，落实水、电、油、气等资源性产品价格改革政策，稳步推进农村土地承包经营权、农村集体建设用地确权登记颁证工作。落实户籍制度改革措施，取消农业和非农业户口，统一登记为居民户口，深入开展户口登记管理专项清理整顿。依法推进商电铝业集团破产工作。四是理顺城区管理体制。优化空间管理范围，将市商务中心区和高铁新站区纳入示范区统一管理，赋予示范区市级经济社会事务管理权限；基本完成产业集聚区与乡镇行政区域管理套合；探索城市建设管理体制，推行网格化城市管理。五是强化创新驱动发展。推动“大众创业、万众创新”，各县（区）都规划建设了电商产业园，一批“双创”平台正在形成。实施科技创新主体培育工程，安排各类科技计划项目89项，争取省重大科技专项3项，新增高新技术企业7家、省级创新团队3家、省级工程技术研究中心9家。梁园、虞城产业集聚区申报省级高新区，已通过省科技厅评审。

（五）基础支撑能力不断增强。一是打造综合交通体系。郑徐客专加快进行，商合杭客专开工建设，连霍高速商丘段拓宽改造已经完成，商登高速建成通车，郑民高速加快推进。商丘机场工程可行性研究报告已上报国家发改委。推进干线公路升级改造，实施农村公路“三年行动计划”，提升道路畅通能力。二是加强能源基础支撑。积极推进与中电国际合作，2×35万千瓦民生热电项目开工建设。加快电网升级改造和西气东输豫东支线二期工程建设，积极发展光伏发电、生物柴油等新能源。三是推进开放招商。加强与国内外500强、行业龙头、知名品牌战略合作，成立7个招商组开展专题招商，促成美力达新能源汽车、亿美工具、海航冷藏车等项目顺利签约。全年引进固定资产投资3亿元以上项目46个，总投资446亿元，利用省外资金539亿元。四是加快重点项目建设。开展百日攻坚、重大项目集中开工活动，省市重点项目完成投资990亿元，为年度计划的110％。金锐金刚石产业园、赛琪工业园、电子信息产业园、中原佳海国际商贸城、高铁安置区、古城棚户区改造安置等重大项目顺利推进。

（六）人民生活持续改善。办好省十项民生工程和市十件实事，全市财政民生支出完成306.9亿元，占公共财政支出的80.2％。推进就业工作，城镇新增就业6.5万人，下岗失业人员再就业2.4万人，高校毕业生就业签订率80％以上。不断完善基本医疗、工伤、生育、失业保险市级统筹，健全医疗保险门诊统筹管理机制，社会保险覆盖面稳步扩大。优化城乡基础教育资源配置，新建、改扩建小学22所、初中12所、九年一贯制学校3所。市文化艺术中心、市体育中心、市实验幼儿园开工建设。完善基层医疗卫生服务体系，做好重大传染病、慢性病防控救治和各类突发公共卫生事件应急处置，病媒生物防制通过考核验收。解决了108.6万农村居民和学校师生饮水安全问题。开工建设各类保障性住房3.2万套；全年发放住房公积金贷款13.7亿元，支持职工改善住房条件。实施我市大别山片区区域发展与扶贫攻坚规划，133个贫困村实施整村推进，15.5万人实现稳定脱贫。坚持计划生育基本国策，促进人口均衡发展。加强生态建设，实施“蓝天、碧水、乡村清洁”工程，加大治污减排力度，秸秆禁烧和综合利用取得明显成效，淘汰黄标车1.8万辆，坚决关停了一批污染严重、治理无望的企业。实施林业生态提升工程，完成造林16万亩。加强和创新社会治理，健全四项基层基础制度，依法做好防范处置非法集资工作，全面抓好矛盾纠纷预防化解、社会治安防控和信访维稳工作，进京非访、越级访控制指标居全省前列。抓好安全生产监管和应急救援体系建设，道路交通管理水平进一步提高，亡人火灾事故为零，安全生产形势持续平稳。

（七）民主法制和精神文明建设不断加强。认真开展“三严三实”专题教育，不断巩固党的群众路线教育实践活动成果，持续解决“四风”问题，营造了风清气正的发展环境。依法履行政府职能，坚决执行人大及其常委会决议决定，自觉接受人大监督、政协监督和社会监督，认真办理人大代表建议和政协提案，支持民主党派、工商联和无党派人士参政议政。全面推进依法治市，深入开展法制宣传教育，圆满完成“六五”普法任务。加强依法行政和服务型行政执法，做好行政监察、行政复议、行政执法监督和审计监督。持续加强社会主义核心价值观教育，开展精神文明创建活动，推进社会信用体系建设，社会文明程度不断提升。

高度重视国防教育，不断加强国防后备力量及人民防空建设，深入开展“双拥”工作，推动军民融合深度发展，驻商解放军、武警部队和民兵预备役为地方发展作出了重要贡献。工会、共青团、妇联等人民团体在经济社会发展中发挥了不可替代的作用，民族宗教、外事侨务、对台工作全面加强，统计、气象、地震、人防、农机、档案、史志等工作取得了新成绩。

各位代表，2015年各项任务的圆满完成，也标志着“十二五”规划主要目标的胜利实现。五年来，我市经济社会发展取得的成就集中体现在以下几个方面：

这五年，综合实力不断攀升。生产总值五年新增660亿元，年均增长10.2％，高于全国全省平均水平；一般公共预算收入较“十一五”末增长1.6倍，年均增长20.8％；全社会固定资产投资累计超过6500亿元，是“十一五”时期的2.3倍，年均增长18.6％。

这五年，产业结构不断优化。2015年，全市三次产业结构为20.9∶42.2∶36.9，二三产业比重较“十一五”末提高5.2个百分点。六大高成长产业、高新技术产业增加值增长较快，现代物流、电子商务、金融保险和文化旅游等发展迅速，信息基础设施逐步完善，农业现代化水平持续提升，粮食总产连年稳定在120亿斤以上（含永城），连续三年被评为“全国粮食生产先进市”。

这五年，改革开放不断深入。推进简政放权和行政职能转变，完成政府机构和事业单位分类改革，取消、下放一大批行政审批事项，国有企业、农村、商事等改革取得积极进展。深化文化体制改革，我市被评为“全国文化体制改革工作先进地区”。对外开放进入历史最好时期，成功举办中国·商丘国际华商节，口岸、保税物流中心等开放平台建设进展顺利，累计利用省外资金2177.5亿元，年均增长20.1％，承接产业转移示范市建设取得明显成效。

这五年，民生保障不断增强。坚持每年办好“十件实事”，全市民生支出占财政支出比重达80％。城乡居民收入年均增长11.2％、13.2％。五年城镇新增就业39.7万人，下岗失业人员再就业12万人，农村劳动力转移就业38万人。各级各类教育质量提升、规模扩大。城乡居民医疗保险、养老保险基本实现全覆盖，保障标准持续提高。建成一大批保障性住房，住房保障体系初步形成。生态环境建设持续加强，节能减排目标圆满完成。

这五年，商丘形象不断提升。诚信商丘建设扎实推进，“商丘好人”群体不断扩大，21人荣登“中国好人榜”，宋静荣获南丁格尔奖。城乡建设成效显著，城镇化率达到47％，五年增加9.2个百分点。我市先后获得“中国优秀旅游城市”、“国家园林城市”、“全国科技进步先进市”、“全国绿化模范城市”、“全国双拥模范城市”和“河南省卫生城市”、“河南省创建文明城市先进市”等荣誉称号。

各位代表！

“十二五”时期是商丘发展进程中很不平凡的五年，也是务实重干、奋发有为的五年，我市经济社会发展又迈上了新台阶、进入了新阶段。这些成绩的取得，是省委、省政府正确领导的结果，是全市上下齐心协力、顽强拼搏的结果。在此，我代表市人民政府，向全市人民、驻商解放军、武警消防官兵和公安民警致以崇高的敬意！向支持政府工作的各位代表、各位委员和社会各界人士表示衷心的感谢！

回顾“十二五”时期的发展，我们主要坚持做到了以下几点：一是坚持发展为先。发展是解决商丘所有问题的关键。我们立足商丘实际，始终把发展作为第一要务，坚持以经济建设为中心，一心一意谋发展，在加快发展中实现经济转型，推进更高质量的发展。二是坚持优化结构。针对经济发展中不全面、不协调、不可持续的问题，以市场需求为导向，调整优化产业结构和产品结构，巩固发展现代农业，改造提升传统产业，大力发展战略性新兴产业，积极发展现代服务业，新型城镇化、新型工业化、农业现代化和信息化的发展空间持续拓展。三是坚持改革创新。把改革创新作为破解瓶颈制约的根本之策，通过政府职能转变、体制机制创新、优化审批流程和深化土地、户籍、国有企业等领域的改革，努力解决阻碍经济发展的重点难点问题，增强了科技创新能力和经济发展活力。四是坚持培育优势。积极适应新常态，立足实际，主动作为，扬长避短，加快实施重大交通项目，增强区位交通优势；发展壮大特色产业，增强产业竞争优势；大力发展现代物流业和电子商务，增强商贸物流优势。五是坚持开放引领。始终把扩大开放作为应对复杂局面、促进改革发展最有效的综合性举措，坚定不移实施开放带动战略，大力发展外向型经济，积极引进行业龙头企业和产业链配套企业，壮大产业集群。六是坚持务实重干。突出问题导向，把握工作重点，脚踏实地做实事、真心实意办好事，不搞短期行为、不做表面文章，做到谋事要实、创业要实、做人要实，在全社会营造了实干为荣、干事创业的良好氛围。

在肯定成绩的同时，我们也清醒认识到，经济社会发展还存在不少困难和问题：一是产业结构总体低端化还没有根本改变，传统产业链条短，自主创新能力弱，质量效益不高，综合竞争力不强；固定资产投资增长放缓，电解铝、煤炭、化工等产品市场价格仍处于低谷，部分行业生产经营仍然困难；财政收入增速放缓，教育、医疗、社会保障、增加工资等刚性支出增长，财政收支矛盾比较突出。二是统筹城乡发展任重道远，城镇化水平不高，中心城区辐射带动能力不强，城乡居民收入差距仍然较大。三是资源环境约束趋紧，资金、土地、劳动力等瓶颈问题依然存在，生态环境总体上仍处于破坏与保护、污染与治理相持时期，安全生产和非法集资监管任务较重。四是发展任务艰巨，区域竞争日益激烈，与省内先进市及周边城市相比，部分指标差距还在拉大。五是作风建设有待加强，一些干部领导方式、工作方法与新形势新任务的要求还不相适应，一些部门办事效率低，懒政、怠政现象依然存在。对于这些问题，我们将高度重视，认真加以解决。

二、“十三五”发展总体思路和奋斗目标

市委四届十三次全会通过的《中共商丘市委关于制定国民经济和社会发展第十三个五年规划的建议》，是指导商丘今后一个时期发展的行动纲领。“十三五”时期，我市的战略定位是：豫鲁苏皖四省接合部区域性中心城市、全国流通节点城市、全国交通枢纽城市、国家历史文化名城、中原经济区承接产业转移示范市。到2020年，全面建成小康社会，经济实力显著增强，人民生活更加殷实富裕，生态环境更加优美宜人，社会大局更加和谐稳定，在区域竞争中赢得主动，在全国、全省发展格局中发挥重要作用。

“十三五”时期是可以大有作为的重要战略机遇期，是全面建成小康社会的决胜阶段，中部地区将成为我国扩大内需的主战场和经济发展的新引擎，经济发展进入速度变化、结构优化、动力转换的新常态，经济社会仍保持总体向好的发展趋势。机遇难得，抓住用好，就能实现跨越赶超；挑战客观存在，应对得当，就能转化为新的契机。我们必须用新的发展思路引领发展行动，准确把握重要战略机遇期的深刻变化，既要增强机遇意识、攻坚意识，又要增强危机意识、紧迫意识，抢抓机遇，乘势而上，脚踏实地，埋头苦干，坚定不移把商丘发展不断推向新的阶段。

今后五年我市经济社会发展的指导思想是：高举中国特色社会主义伟大旗帜，全面贯彻党的十八大和十八届三中、四中、五中全会精神，以马列主义、毛泽东思想、邓小平理论、“三个代表”重要思想、科学发展观为指导，深入贯彻习近平总书记系列重要讲话精神，按照“五位一体”总体布局和“四个全面”战略布局，坚持发展第一要务，牢固树立创新、协调、绿色、开放、共享的发展理念，以提高发展质量和效益为中心，加快形成引领经济发展新常态的体制机制和发展方式，统筹推进经济建设、政治建设、文化建设、社会建设和生态文明建设，全面深化改革，扩大对外开放，全面建成小康社会，建设经济强、城乡美、活力足、社会安、生态好、百姓富的魅力商丘，为中原更加出彩作出更大贡献。

今后五年我市发展的蓝图和愿景是：

——经济实力显著增强。到2020年，地区生产总值和城乡居民人均收入比2010年翻一番以上，全面建成小康社会。在提高发展平衡性、包容性、持续性的基础上，继续保持经济平稳较快增长，主要经济指标增速高于全省平均水平，一般公共预算收入与经济增长同步。

——转型升级取得重要进展。创新能力大大提升，工业化信息化融合发展水平明显提高，过剩产能化解取得成效，新增长点形成规模，消费对经济增长贡献明显加大，农业现代化取得明显进展，服务业拉动作用明显增强，战略性新兴产业占比大幅提高，产业迈向中高端水平，发展质量和效益明显提升。

——改革开放实现重大突破。重要领域和关键环节改革取得决定性成果，形成科学规范、运行有效的体制机制，改革红利充分释放，发展活力不断增强。对外开放深入拓展，内陆开放高地加快形成，承接产业转移示范市建设成效扩大，参与国际经济合作能力和水平明显提升，成为“一带一路”具有影响力的城市。

——城乡面貌焕然一新。城镇化进程进一步加快，城镇功能和服务能力明显提升，城乡发展一体化水平明显提高，全市常住人口城镇化率明显提高，中心城市辐射力带动力明显提升，市政基础设施更加完善，市文化艺术中心、体育中心和市民服务中心投入使用，美丽乡村建设取得明显成效。

——生态环境质量持续改善。污染治理和生态修复实现重大突破，空气质量明显好转，生产方式和生活方式绿色、低碳水平上升。单位生产总值能耗下降，二氧化碳和主要污染物减排总量控制在国家下达的指标内，土地、水等资源利用效率大幅提高，平原绿化继续保持全省先进位次，中国“长寿之乡”称号进一步叫响，可持续发展水平不断提升。

——人民生活水平和质量全面提高。城乡居民收入高于经济增长，现行标准下农村贫困人口实现脱贫，贫困县全部摘帽。人民群众拥有更好的教育、更稳定的工作、更满意的收入、更可靠的社会保障、更高水平的医疗卫生服务、更舒适的居住条件、更优美的环境，社会事业全面发展，社会更加和谐稳定。

《商丘市国民经济和社会发展第十三个五年规划纲要（草案）》已印发给各位代表，请一并审议。

我们相信，有省委、省政府和市委的正确领导，有全市九百万人民的团结奋进、顽强拼搏，我市“十三五”发展的宏伟蓝图一定能够变成美好的现实！商丘发展一定能够实现新跨越、再铸新辉煌！

三、2016年发展目标和重点工作

今年是实施“十三五”规划、全面建成小康社会决胜阶段的开局之年，也是推进结构性改革的攻坚之年。做好今年的政府工作，对于落实市委四届十三次全会和市委经济工作会议精神，推动全市经济社会持续健康发展，全力构筑中原经济区东部战略支撑具有重要意义。

今年经济社会发展的主要预期目标是：全市生产总值增速8.5％左右；一般公共预算收入与经济增长同步；全社会固定资产投资增长15％；社会消费品零售总额增长12％；外贸出口增长8％左右；实际利用外商直接投资增长8％左右；城乡居民人均收入与经济增长同步；居民消费价格涨幅控制在3％左右；环保、节能减排等约束性指标完成省定目标。

围绕上述目标，重点做好六个方面工作：

（一）推动产业结构优化升级，提高经济发展质量和效益。我市自身结构性矛盾比较突出，必须落实好供给侧结构性改革，加快产业结构调整，重点围绕去产能、去库存、去杠杆、降成本、补短板五大任务，优化要素配置，提高供给体系的质量和效率。

推进工业转型升级。一是提升产业发展质量。市级加快培育装备制造、食品工业、纺织服装制鞋等超千亿级产业集群，县域培育形成12个超百亿级产业集群。改造提升传统产业，重点围绕制冷装备、食品工业、纺织服装制鞋、电动车等传统产业，引导企业升级改造，加快建设“三城五基地”。不断推进战略性新兴产业，重点发展电子信息、生物医药、新材料、环保装备等产业，积极发展新能源汽车，推动电动车产业结构优化和产品升级换代。实施质量强市战略，培育一批拥有自主知识产权和知名品牌的优势企业。对没有市场、长期亏损停产半停产企业，要有序实施市场出清。二是强化企业服务。落实国家降低制造业增值税税率、职工社会保险社会缴付比例、电价等政策，清理规范中介服务收费和企业贷款中的不合理收费，切实减轻企业负担，帮助企业降低交易、人工、物流、财务、电力等成本。加强对经济形势的研判，做好煤电油气运、土地、用工等要素保障，确保生产要素向重点产业、重点企业、高成长性企业和产业集聚区倾斜。深化产销对接，组织筹办好2016年电动车、纺织服装制鞋、食品、制冷四个展会，帮助企业开拓市场。完善信用担保体系，用好工业企业还贷应急周转金，开展多层次银企对接，建立完善银行对企业抽贷报告制度，防范资金链断裂风险。完善中小企业公共服务体系，落实好扶持中小微企业发展的政策措施，争取5家企业在新三板挂牌。三是推进工业化和信息化融合。落实《中国制造2025》，加快从区域、行业、企业推进工业化和信息化深度融合。加快信息基础设施建设，实施“互联网＋”行动计划，扩大“光网城市”建设成果。加快工业智能改造升级，开展“智慧园区”建设，引导企业利用信息技术改造传统工艺和生产流程，建设“智能工厂”和“智能车间”，实现信息技术在传统制造业的全面渗透和深度融合。四是加快产业集聚区建设。推动产业集聚区提质转型创新发展，重点引进智能装备、高档服装面料及制鞋、生物医药、运动健康、中高端食品、电子信息、新能源汽车等产业，大力承接龙头企业和全产业链转移，力争全年落地亿元以上项目80个，建成投产项目80个。加快集成配送、仓储加工、展示交易、检验检测、人力资源等服务平台建设，加快教育、医疗卫生、住房等公共服务能力建设，培育创建一批国家、省级知名品牌示范产业集聚区。2016年全市产业集聚区完成固定资产投资突破1200亿元；规模以上工业增加值达到450亿元、主营业务收入达到1900亿元。

大力发展现代服务业。一是发展高成长性服务业。以豫东综合物流集聚区为平台，大力发展现代物流业，加快物流园区、配送中心和现代市场体系建设，推动中心城区市场规划、搬迁和转型升级；加快发展基础信息传输、电子商务、云计算和物流网等信息服务业，建设商丘大数据云计算中心。二是抓好服务业“两区”建设。积极引进楼宇经济、总部经济，发展信息咨询、科技研发、金融服务和健康服务、教育培训、居家养老等新兴服务业，加快新建项目集中布局，力争全市服务业“两区”完成年度投资80亿元。三是建设电子商务示范市。加大政策支持力度，落实好政府奖励政策，引导阿里巴巴·商丘产业带发展壮大，力争2016年底入驻企业突破1200家。与阿里巴巴在互联网金融保障、电商人才培训、电商业务、电商生态服务等方面开展深度合作，打造集电商主体、综合服务、人才支撑为一体的电子商务生态链。开展跨境电子商务，实现跨境电商营业额翻番。开展电子商务示范创建工作，创建一批省级示范园区、示范企业和国家级电子商务进农村综合示范县。四是大力发展文化旅游业。积极发展生态、文化、工业、休闲、康养等旅游业态，加快推进商丘古城、黄河故道等重点旅游资源开发，推进民权王公庄创建国家4A级景区，酥梨生态景区、容湖景区、白云禅寺景区、鑫广家庭农场创建国家3A级景区。

促进农业结构调整。一是提高粮食综合生产能力。推进高标准粮田“百千万”工程，新建50万亩高标准粮田，将高标准粮田培育成多种形式适度规模经营示范区、农业发展方式转变先导区和现代化粮食生产核心区。二是培育现代农业产业集群。发展粮食深加工产业化集群，做强一批龙头企业，打造一批农产品知名品牌，发展一批农产品批发市场。积极发展蔬菜、果品、渔业、花卉等都市生态农业。强化畜牧产业化集群发展，建设全省重要的优质畜产品生产加工基地，每个县（区）重点培育1—2个现代畜牧产业化集群。三是深化农村改革。完成农村土地承包经营权确权登记颁证工作，基本完成农村集体建设用地使用权确权登记发证工作。鼓励引导农户依法采取转包、出租、互换、入股等方式流转承包地，积极探索土地托管、土地银行等土地流转方式。制定财政、金融、保险、用地、人才等扶持政策，推动新型农业经营主体参与财政支农项目建设、运行和管理。抓好国有农场、林场、供销社改革，推进农村信用社改制组建农村商业银行。

（二）努力扩大有效需求，增强经济发展拉动力。从供需两端同时发力，以创新供给带动需求扩展，充分发掘消费潜力，培育新的消费热点，增强居民消费能力，实现稳增长和调结构互为支撑、互促共进。

扩大有效投资。把重大项目建设作为扩大投资需求的重要抓手，今年初步安排省市重点项目243个，计划完成投资1010亿元。一是加强交通枢纽项目建设。进一步做好郑徐客专、商合杭客专配套市政工程建设，保障郑徐高铁投入运营、商合杭高铁加快建设。支持郑民高速民权段建设，做好连霍高速豫皖界至商丘段改扩建工程前期工作；积极推进干线公路升级改造，加快实施农村公路“三年行动计划”，提高公路通达能力；争取开工建设商丘机场、沱浍河航运工程夏邑段项目。二是加强能源项目建设。推进与中电国际合作，加快2×35万千瓦民生热电联产项目建设，开工建设中电国际垃圾发电项目。推进电网升级改造，建设现代化坚强智能电网，进行新一轮农村电网升级改造。积极发展以天然气、生物燃料、电力等为主的新能源动力汽车。三是加强水利基础设施建设。实施引江济淮工程和惠济河平原洼地治理工程，完成任庄及马楼水库除险加固、三义寨引黄灌区续建配套与节水改造、小农水重点县项目建设。启动农村饮水安全提升工程，提高集中供水率、自来水普及率、水质达标率和供水保证率。四是服务好重点项目建设。采取联审联批、领导联系项目、目标责任制、部门联动配合等措施，加快推进项目进度。对中央预算内投资项目和国家专项建设基金项目，积极创造条件，按时开工建设。围绕我市发展的薄弱环节，加强重大项目谋划储备，争取更多项目纳入国家和全省盘子。

扩大消费需求。推动居民消费结构升级，大力发展住房、汽车消费，增强对消费的带动作用。积极培育通信、旅游、休闲、健身等消费热点，发展健康养老、医疗保健、教育培训、家政等服务消费，引导文明健康的文化娱乐消费，健全便民、安全、快捷的商业网络，发展信贷消费、网上购物、租赁消费等新的消费方式，促进假日和节庆消费。扩大农村消费，推进农村商贸流通和市场体系建设，引导鼓励城市大型商贸流通企业经营网络向农村延伸，全面开展电子商务进农村活动，2016年年底覆盖我市80％的行政村。

扩大房地产需求。正确引导房地产消费，出台棚户区货币化安置政策，提高棚户区货币化安置率，鼓励通过消化存量商品房安置棚改居民，完善农民进城购房政策，鼓励自然人和各类机构投资者购买库存商品房，消化库存，满足新市民住房需求。今年不再新建公租房，通过发放货币补贴、由政府购买存量商品房解决低收入家庭住房问题。

扩大出口规模。引导外贸企业转型升级，优化出口商品结构，培育出口品牌，提升外贸核心竞争力、抗风险能力和可持续发展能力。推动优势企业开展对外直接投资、对外承包工程、对外劳务合作，带动设备出口。培育年出口超千万美元的企业6家、500万美元以上的企业15家，有进出口实绩的企业超过190家。扩大农产品出口，建成出口食品农产品质量安全示范区6个以上。

（三）加快推进新型城镇化，构筑城乡一体化发展格局。统筹推进“一心一廊两轴五组团”建设，2016年，全市城镇化率达到49％左右，城镇人口达到360万左右。

加快中心城区建设。完成《商丘市城市总体规划（2015—2030）》和中心城区25平方公里控制性详细规划编制，实现中心城区控规全覆盖；严格规划实施，切实维护规划的严肃性和权威性。重点实施高铁商贸区、商务中心区和古城文化区建设，加快推进商虞一体化进程，统筹做好城区道路、园林绿化、市容环卫、供排水、停车场等城市基础设施建设。实施日月湖、河道、公园和道路绿化，确保2016年年底中心城区绿化覆盖率达到40％。继续实施城区内河水系治理，加快推进污水处理厂和污水管网建设，实施护坡绿化、桥涵闸坝等工程建设，打造岸绿、水清、景美的生态廊道。以“六城”联创为抓手，持续推进城市网格化管理，重点加强交通秩序、沿街商店、流动商贩、施工工地和建筑垃圾管理，建立市容市貌管理长效机制，提高环卫机械化清扫率，倡导全民参与城市管理，共创美好家园。

夯实城乡发展基础。强化5个县城组团发展，实现产业集聚区、商务中心区和特色商业区与县城主城区融合发展，提升综合承载能力，提高县城品位。以24个重点镇为示范点，带动全市小城镇快速协调发展，努力形成特色鲜明、产业配套、功能完善的小城镇体系。抓好4个全省经济发达镇行政管理体制改革试点工作。稳步推进新农村建设，统筹抓好农村基础设施建设、村庄规划和人居环境综合整治，改善农村生产生活条件。

加快城乡一体化发展。坚持产城融合、以产促城，把产业支撑作为推进城乡一体化发展的重要基础，围绕主导产业，在二三产业集聚化、高端化发展基础上，加快城镇基础设施和公共服务设施向农村延伸，推动都市生态农业发展，引领城乡产业转型升级。发挥市城乡一体化示范区的示范引领作用，重点推进传化物流港、特色商业街、万达广场等产业项目，城市森林公园、日月湖景观、日月河、学校、医院等社会事业项目，长江路跨京九铁路桥、畅通路、华商大道东延等基础设施项目，启动商合杭新区站规划建设，为全市城乡统筹发展提供借鉴。

加快促进农业转移人口市民化。落实好农民进城购房补贴政策，加快落实户籍制度改革和居住证制度，做好进城落户农民的社会保障、就医就业、子女入学等方面的基本公共服务，维护好进城落户农民土地承包权、宅基地使用权、集体收益分配权，解决农民落户城镇后顾之忧。

（四）持续深化改革开放，释放经济社会发展活力。加大改革创新力度，促进土地、资本、技术、人力等要素优化配置，释放经济发展活力；实施“东引西融”，积极对接“一带一路”战略，努力扩大承接产业转移示范市成效。

深化各项改革。落实市委全面深化改革的实施意见，扎实推进以经济体制改革为主的各领域改革。深化行政审批制度改革，激发市场活力和社会创造力。建成公共资源交易平台，实现公共资源在统一规范的平台上交易。完善财税制度改革、国有企业改革，支持发展混合所有制经济。实施国有企业负责人薪酬制度改革，深化机关事业单位工资收入分配制度改革，落实县以下机关公务员职务与职级并行制度，加大工资政策向基层倾斜力度。

大力实施开放招商。一是继续招大引强。重点加强与行业100强企业的联系对接，力争引进行业龙头企业30家以上，引进投资3亿元以上项目50个以上。每个县（区）引进符合主导产业的3亿元以上项目2个、5亿元以上项目2个，力争引进10亿元以上项目1个。二是完善产业链条。围绕制冷装备、纺织服装制鞋、电子信息、金刚石超硬材料、农资化工、五金工量具、新能源汽车、生物医药等主导和特色产业，以产业链整合提升、促进产业集聚为重点，突出延链补链，完善配套能力，拉长产业链条。三是创新招商方式。拓展重点区域，建立珠三角、长三角及港澳地区招商战区，主攻东部沿海发达城市。开展专业招商活动，发挥7个专题招商组的作用，打破市县之间、县区之间的行政界限，开展以商招商、协作招商。精心组织，办好2016中国·商丘国际华商节。四是做好招商保障。加快推进商丘综合保税区前期工作和商丘铁路一类口岸的申报工作，加快建设商丘保税物流中心。与连云港、日照、青岛等沿海港口建立稳定的沟通联络机制，确保货物进出口无障碍、零滞留。做好土地供应、融资服务、并联审批等工作，加强高校、职业院校与招商引资项目的联系，实施订单培养、定向培训，满足企业对技能型人才的需求，确保招商项目引得进、进得来、留得住，发展得好。

深入推进大众创业万众创新。落实扶持政策，加强创新团队和创新人才队伍建设，鼓励金融支持创新创业，用活用好专项扶持资金，支持多元化投资建设科技企业孵化器、大学生创业园、创业服务中心等创业孵化载体，发展创新创业孵化基地，完善创新创业公共服务体系，推动科技、产业有机融合，强化知识产权保护，营造大众创业、万众创新的良好环境。

推动重点领域科技创新。围绕产业集群，加快研发、技术转移转化、科技金融和企业培育等服务体系建设。围绕高端装备制造、生物医药、电子信息、新材料、新能源、新能源汽车等领域，组织实施重点研发专项，支持创新水平高、产业带动强、具有自主知识产权重大科技成果的研发及产业化，力争突破一批关键核心技术。2016年，申报高新技术企业8家以上、省级工程技术研究中心8家以上，启动申报商丘国家高新技术开发区。完善与中科院、央企等创新合作机制，加强与河南大学、商丘师院等高校开展市校合作，支持国内外知名高等院校、科研机构在我市建立研发基地，促进新技术新成果转移转化。

（五）加强生态环境建设，着力保障发展容量。深入实施蓝天、碧水、乡村清洁、城市河流清洁行动，建设生态美丽商丘。

继续实施“三大工程”。实施“蓝天工程”，加大废气排放治理力度，严格机动车环保标志管理，加快淘汰黄标车和老旧车辆。开展城市卫生清扫行动，严格控制城市施工和道路扬尘，积极开展餐饮业油烟污染治理，进一步减少大气污染物排放。实施“碧水工程”，组织开展重点河流环境综合整治，实施城市河流清洁行动计划，推动新一轮城镇污水处理设施建设，确保达到省定目标要求。加强水源地保护，确保饮用水源地水质安全。实施“乡村清洁工程”。持续推进美丽商丘·整洁村镇活动，开展农村环境综合整治，强化畜禽养殖污染防治，改善农村人居环境。积极推进生态文明示范区建设，确保完成省级生态乡镇、生态村创建任务。

大力发展循环经济。围绕化工、钢铁、发电等传统行业，实施清洁生产，建立固废循环利用、中水回用等资源再生利用体系；推进种植业、养殖业、林业、水产业和农副产品加工业之间的循环链接，切实做好秸杆禁烧和综合利用工作，提高农业废弃物资源利用水平。

加强环境监管。围绕主要减排任务的宏观把控，强化年度目标管理，落实污染物排放许可、污染物排放总量控制制度，集中有限指标保障重点项目建设。开展环保专项执法检查，坚决对违法建设、违法生产、违法排污等行为进行查处。

推进林业生态建设。围绕创建国家森林城市目标，重点做好高速公路防护林、生态廊道网络、平原沙荒造林、农田防护林、城镇社区绿化、森林抚育与改造等工程，完成造林任务12.3万亩，森林抚育和改造3.2万亩。抓好国家森林公园、湿地公园、水利风景区建设，做好迎接国家森林城市评审的各项准备工作。

（六）突出民生改善，增强人民群众现实获得感。把保障改善民生作为一切工作的出发点和落脚点，实施好十件实事，增加基本民生支出，健全基本公共服务，实现共享发展。

实施脱贫攻坚。健全精准扶贫、精准脱贫工作机制，拓宽扶贫渠道，在转移就业扶贫、产业扶贫、安居扶贫、基础设施扶贫、保障扶贫等方面，加大财政、金融扶持力度，引导资金、人才、技术向贫困地区集聚。严格落实领导主体责任，完善督导考核机制，创新扶贫工作机制，确保完成年度脱贫任务。

保持就业稳定增长。完善就业创业服务体系，做好大学生就业创业指导，搭建好高校毕业生与用人单位之间的桥梁。加大创业培训力度，以创业带动就业。继续实施全民技能振兴工程，做好农民工技能培训、下岗职工再就业工作。

持续提高社会保障水平。稳妥推进机关事业单位养老保险制度改革，落实职业年金实施办法。做好全民参保登记试点工作，完善灵活就业人员、城镇个体工商户等群体的缴费办法，研究农民工等中断缴费群体的参保措施。完善城乡居民养老、医疗、工伤、生育、失业保险市级统筹，做好被征地农民社会保障，落实省内异地就医即时结算各项衔接措施。

统筹发展各项社会事业。加快第二轮学前教育三年行动计划，按时完成中小学标准化建设任务，促进义务教育均衡发展，普及高中阶段教育。支持社会力量办学，发挥好职业教育和高等教育资源优势，推进职业教育攻坚二期工程，培养适应商丘经济社会发展的各类技能人才。组织做好“舞台艺术送农民”活动，继续开展“百戏进百乡”活动和农村公益电影放映，推进全市基层综合性文化服务中心建设工作。加强公共卫生服务能力建设，积极做好省级卫生城复核和全国卫生城创建工作。落实好全面放开“两孩”政策，加强计生优质服务，提高出生人口素质。

抓好安全生产。严格落实安全生产责任制，深入开展安全隐患大排查大整治，全面加强道路交通、建筑施工、消防、危爆物品、特种设备等重点领域安全监管，严格责任追究，严防发生重特大安全事故。强化食品药品监管，保障人民健康安全。

维护社会大局稳定。完善信访工作机制，畅通人民群众诉求渠道，排查化解社会矛盾，切实维护群众利益。加强社会救助体系建设，提高救助工作水平。切实抓好社会治安综合治理，完善治安防控体系，防范和处置非法集资，依法打击涉嫌违法犯罪行为，增强人民群众安全感。加强重大公共安全事件预警、应急、救援体系建设，完善应急管理体制，强化社会危机管理，不断提高预防和处置突发事件的能力。

深入推进国防教育和国防后备力量建设，拥护和支持国防军队改革，支持驻商解放军和武警部队、预备役部队、民兵和人民防空建设，积极开展双拥共建活动，推动军民融合发展，巩固和发展军政军民团结。支持工会、共青团、妇联等人民团体开展工作，发挥工商联等社会团体作用。做好民族宗教、统计、外事侨务、对台、史志、气象、防震减灾等工作。

办好事关人民群众切身利益的“十件实事”：

1.建设119条城区道路，新建打通迎宾路、北海路、长江东路等74条道路，提升改造黄河路、凯旋路、归德路等45条道路。

2.规划商都公园、花博园、城市森林公园、古宋河畔公园、湿地公园、植物园、野生动物园、汉梁文化公园二期、三陵台公园、沙河公园、清凉寺公园。今年开工建设6个公园。

3.开工建设市一中、市一高、商丘医专新校区和商丘市民服务中心。

4.实施运河、日月河等河道景观带建设，建成投用第六污水处理厂，完成第八污水处理厂主体工程。

5.开工建设市城乡一体化示范区医院、市中心医院儿科病房楼（市儿童治疗中心）、市中医院门诊楼扩建项目。

6.投资1000万元购买纯电动空调公交车、双层观光城市公交车；实施公共自行车项目，购买公共自行车2000辆。

7.筹资2亿元，对农民和其他购房对象购买中心城区普通商品住房实施补贴；筹资3亿元，对符合条件的住房保障对象购买中心城区普通商品住房实施补贴；筹资1.2亿元，对农民和其他购房对象购买县城城区普通商品住房实施补贴。

8.完成100个贫困村的整村推进任务，实现15万农村贫困人口稳定脱贫。

9.投资10亿元，新建农村公路1000公里。

10.投资5.5亿元，实施城乡电网改造。

四、切实加强政府自身建设

做好今年政府工作，必须以建设法治政府、服务型政府为重点，巩固深化党的群众路线教育实践活动和“三严三实”专题教育成果，推进政府自身改革建设，提升政府公信力和执行力。

（一）坚持依法行政。认真落实全面推进依法治国战略，忠实履行宪法和法律赋予的职责，做到法无授权不可为、法定职责必须为。加强法治培训教育，领导干部带头学法尊法用法，行政执法人员要强化依法行政意识、制度规则意识、权责对等意识，提升运用法治思维和法治方式推进依法行政的能力。完善办事规则制度，建立健全首问责任制、行政执法纠错制、行政过错责任追究制等制度，规范政府职权。坚持依法行政，规范执法行为，探索实行跨部门综合执法，减少政府执法队伍种类，加大事关重点领域执法力度，维护群众合法权益。拓宽监督渠道，自觉接受人大工作监督和法律监督，接受政协民主监督，接受社会监督和舆论监督，保障权力依法、公开和公正运行。

（二）坚持科学施政。完善决策程序，重大行政决策由公众参与、专家论证、风险评估、合法性审查、集体讨论决定，落实决策跟踪反馈、决策监督，确保民主决策、科学决策。善于改革创新，研究新情况、解决新问题、总结新经验，及时调整、完善发展规划和工作思路。牢固树立宗旨意识，坚持问政于民、问需于民、问计于民，忠实履行服务群众、改善民生职责，多做打基础、利长远、惠民生的事，全心全意为人民服务。严格决策实施，认真贯彻执行中央、省委省政府和市委决定，落实政策不走样，一张蓝图绘到底。

（三）坚持从严治政。全面加强公务员队伍思想政治建设和作风建设，从严治政，正风肃纪，加快形成风清气正、干事创业、遵纪守法的良好政治生态。深入开展“不想为、不能为、不敢为、不作为”专项治理，倡导求真务实工作作风，进一步转变工作方式，勇于担当，主动作为，不动摇、不折腾，办实事、求实效，提升政府效能和执行力。完善科学绩效考评体系，对实绩突出的先进典型进行表彰奖励，对影响工作、贻误发展的坚决惩处。

（四）坚持廉洁从政。严格遵守政治纪律和政治规矩，认真落实中央八项规定和省市有关纪律要求，厉行勤俭节约，反对铺张浪费，坚持不懈抵制“四风”。完善惩治和预防腐败体系，认真落实党风廉政建设责任制，遵守廉政准则，切实履行“一岗双责”，保持清廉本色。完善财政资金支出、工程项目招投标、国有资产交易等各项制度，扎牢制度笼子，坚决打掉寻租空间，努力铲除腐败土壤，从源头上预防腐败，以廉政建设和反腐败斗争实效保障发展、取信于民！

各位代表！

人民赋予重托，奋斗创造未来。让我们在市委的坚强领导下，紧紧团结和依靠全市人民，锐意进取，开拓创新，共同创造商丘更加美好的明天！
